# Knowledge gaps and practices for glaucoma among adults in Palestine: a cross-sectional study

**DOI:** 10.3389/fopht.2026.1953940

**Published:** 2026-10-05

**Authors:** Mohammad W. Awlad Mohammad, Basel Bader, Mohammad Fuad Sayes, Alhareth M. Amro, Toqa W. Awlad Mohammad, Essa Hasheem, Mohammad Amro, Darwish M. Mosameh, Dania Amwas, Abdallah Qawasmeh, Farid k Abu Sham’a

**Affiliations:** 1 Faculty of Medicine, Al-Quds University, Jerusalem, Palestine; 2 Faculty of Medicine, Palestine Polytechnic University, Hebron, Palestine; 3 Faculty of medicine, Mansoura University, Mansoura, Dakahlia, Egypt; 4 Department of Medicine, College of Medicine, Hebron University. Hebron, West Bank, Palestine; 5 Health Education and Scientific Research Unit, Ministry of Health, Ramallah, Palestine

**Keywords:** attitudes, awareness, eye health, glaucoma, knowledge, Palestine, public health

## Abstract

**Background:**

Glaucoma is a leading cause of irreversible blindness globally, often progressing asymptomatically until advanced stages. Awareness and knowledge of glaucoma are critical determinants of early diagnosis and treatment-seeking behavior. Data on glaucoma awareness in Palestine are scarce. This study aimed to assess the level of awareness, knowledge, and attitudes regarding glaucoma among the general Palestinian population and to identify sociodemographic factors associated with inadequate knowledge.

**Methods:**

A population-based cross-sectional study was conducted from December 1 to December 30, 2025, using an online questionnaire distributed to adults (≥18 years) residing in the West Bank and Jerusalem. The questionnaire included sociodemographic information, glaucoma awareness, knowledge, attitudes, and practices. Reliability was assessed using Cronbach’s alpha (α = 0.87). Knowledge scores were calculated with a maximum of 8 points; good knowledge was defined as ≥6 correct answers. Data were analyzed using SPSS v23, employing chi-square tests and multivariable logistic regression.

**Results:**

A total of 741 participants completed the survey (50.2% male, 49.8% female). Overall, 54% had heard of glaucoma, but only 13.8% demonstrated good knowledge. Prior awareness of glaucoma was the strongest predictor of good knowledge (aOR = 7.89, p < 0.001). Knowledge gaps were notable regarding the asymptomatic nature of early disease (20.9%), irreversible vision loss (57%), and key risk factors including age ≥40 years (49%), family history (44.9%), and diabetes (55.1%). Older adults, rural residents, and individuals with lower educational levels had significantly lower knowledge scores. Only 26.9% of participants reported eye examinations within the preceding year, and screening intentions were associated with better knowledge (p = 0.015).

**Conclusions:**

Although awareness of glaucoma is moderate in Palestine, comprehensive knowledge remains low, particularly among high-risk populations. Public health interventions should target education beyond name recognition, emphasizing asymptomatic progression, irreversible vision loss, and risk factors. Integrating glaucoma education into clinical visits and community-based programs may improve early detection and prevent blindness.

## Introduction

The key to preventing many diseases is early detection and diagnosis. One of the most significant of these conditions is glaucoma. Glaucoma is a chronic optic neuropathy characterized by progressive optic nerve damage and corresponding visual field loss, commonly associated with elevated intraocular pressure (1, 2). the second leading cause of blindness worldwide and a leading cause of irreversible blindness, with 3.61 million blind individuals and more than 4 million visually impaired people globally (2). Despite advances in diagnosis and treatment, glaucoma continues to impose a substantial public health burden, largely due to its insidious onset and asymptomatic progression in early stages.

Vision loss from glaucoma is irreversible, and delayed diagnosis often results in permanent blindness. Alarmingly, in a Congolese study, recent clinical evidence indicates that up to 36% of patients present with blindness in at least one eye at their first ophthalmic consultation, reflecting advanced disease at the time of diagnosis (3). This late presentation is frequently attributed to poor awareness, limited knowledge, and inadequate recognition of early symptoms, which delay treatment-seeking behavior (4).

People’s treatment-seeking behavior is significantly influenced by their awareness and knowledge of glaucoma. The lack of awareness is pervasive throughout the world and is not confined to any one location; Asian Pacific Glaucoma Society Guidelines report “up to 36% of those with Primary Open-Angle Glaucoma (POAG) and 70% of those with Primary Angle-Closure Glaucoma (PACG) were blind at the time of examination in population-based studies” (5). The percentage of knowledge and awareness was significantly low in a study conducted in Syria (6). A similar result was found in prior research conducted in Africa, which revealed that Nigerians and Ghanaians had low levels of awareness and understanding of glaucoma (7).

Additionally, there aren’t many studies assessing awareness of eye diseases in general, and glaucoma in particular, in the Middle East, where Saudis and Jordanians have high awareness of glaucoma but low to average knowledge of it (8, 9). For this reason, it was necessary to conduct awareness assessment studies in additional countries in the region, such as Palestine, Lebanon, and others.

Despite the recognized importance of public awareness, data on glaucoma knowledge, awareness, and attitudes in Palestine remain scarce. Given the unique healthcare challenges and limited screening opportunities in the region, understanding population-level perceptions of glaucoma is essential for designing targeted educational and preventive interventions.

Therefore, this study aims to assess the level of awareness, knowledge, and attitudes regarding glaucoma among the general population in Palestine and to identify sociodemographic factors associated with inadequate knowledge. The findings are intended to inform public health strategies aimed at promoting early detection, improving treatment-seeking behavior, and ultimately reducing glaucoma-related visual impairment and blindness.

## Methods

### Study design and setting

This cross-sectional study was conducted in the West Bank and Jerusalem in Palestine from December 1, 2025, to December 30, 2025, utilizing an online questionnaire created with Google Forms, which was distributed through various social media websites. The questionnaire was specifically designed for data collection and included demographic inquiries and items about glaucoma awareness among Palestinian adults.

### Instrument design and validation

After a section providing information about the research goals, confidentiality, and consent, there is a section to provide electronic consent, and then the questionnaire consists of four parts: sociodemographic or background information, consisting of 7 questions; awareness and information sources, with one closed question and one additional question with multiple choices specifying the answer; knowledge and assessment questions to measure clinical characteristics and awareness of glaucoma, with 8 questions with (True) or (False) or (not sure) responses; and attitudes & practices, in 3 questions. We have allocated one point (1) to each question from the third questionnaire group for each correct answer and zero points (0) to incorrect or “Not sure” responses.

The questionnaire underwent forward and backward translation. The data-gathering method employed an online questionnaire derived from a prior study (9). The co-authors initially prepared it in English. It was rendered into Arabic (suitable to the local accent), then a pilot study was done with 30 responses, and the knowledge scale shows good internal consistency (Cronbach’s α = 0.87) (10).

### Study population and sampling

Our population included the Palestinian public population who are older than 18 years old. No educational restriction. Non-probability, voluntary response sampling was used for our study.

The use of voluntary response sampling facilitated swift, multi-institutional recruitment throughout the West Bank and Jerusalem, enhancing feasibility and geographic coverage.

### Inclusion and exclusion criteria

Our inclusion criteria were Palestinian adults aged 18 years or older. There were no educational restrictions, except that participants needed to be able to read, as they were expected to answer the questionnaire on their own and give electronic consent to participate. Exclusion criteria include non-Palestinian adults or those younger than 18 years old; otherwise, no specific exclusion criteria were present.

### Sample size calculation

The sample size was calculated using the formula for estimating proportions in an unlimited (infinite) population (11), as the target population comprised a large number in the country. Assuming a 95% confidence level (Z = 1.96), a 5% margin of error, and a conservative response distribution of 50% to maximize variability, the minimum required sample size was calculated to be 384 participants. As this study only included participants with complete questionnaire data, the recruitment target was set at 384. The final analytic sample of 741 participants substantially exceeded this requirement.

### Ethics

Ethical approval with a ref. no. 673/REC/2026 was sought from the Al Quds University Ethics Committee. Confidentiality, anonymity, and voluntary participation were ensured. Informed consent was obtained from all participants. Data were exclusively managed by the authors. All authors who handle the data will keep it private and confidential.

### Statistical analysis

The data were automatically exported from Google Forms to Excel, and analysis were performed using the Statistical Package for Social Sciences software package (SPSS Inc., Chicago, IL, USA), version 23. Means and standard deviations (SD) are employed to represent continuous variables, while frequencies and percentages are utilized to convey the data. The chi-square test was employed to evaluate bivariate associations between categorical variables. Multivariable logistic regression was used to estimate adjusted associations with good knowledge.

Knowledge scores ranged from 0 to 8. Two score-based sensitivity definitions were evaluated: Good75 (score ≥6/8, ≥75% correct) and Good80 (score ≥7/8, ≥87.5% correct). The Good80 label denotes the ≥80% threshold, which requires at least seven correct answers on an eight-item scale, which will be described using “Strict good knowledge,” term, these cutoffs were established from published literature (8).

For the strict sensitivity analysis, a reduced multivariable logistic regression model was used to limit model complexity in view of the 60 outcome events. Responses of “not sure” and “not necessary” to the screening-importance item were combined and compared with “very important.

## Results

Non-probability, voluntary response sampling was used for our study; the questionnaire was sent to a large number of our population through private messages and large groups, which makes it impossible to calculate the response rate. A total of 741 respondents were included. Participant characteristics and key eye-health practices are summarized in **Table 1**. The sample was nearly balanced by gender (male: 372 [50.2%], female: 369 [49.8%]) and was predominantly young, with 18–24 years representing 49.9% of participants. Most respondents reported a bachelor’s degree (62.1%), and 61.3% lived in urban areas. A history of chronic disease was reported by 12.1%. Regarding eye-health behaviors, 64.9% reported ever having had a comprehensive eye examination by an eye-care professional, and the timing of the last eye check-up varied (including 23.5% who reported never having had a comprehensive eye check-up). Most participants indicated willingness to follow a provider’s recommendation for an eye examination, and the majority considered glaucoma screening for adults over 40 years to be “very important” (**Table 1**). Overall, 54.0% of participants reported having heard of glaucoma, while 46.0% had not. Among those who had heard of glaucoma, information sources commonly included online platforms and interpersonal sources, with healthcare professionals and academic settings also frequently reported.

**Table 1:** Sociodemographic characteristics and eye-health practices of study participants (N = 741).

Variable	Category	n	%
Gender	Male	372	50.2
	Female	369	49.8
Age	18-24	370	49.9
	25-34	176	23.8
	35–44	101	13.6
	45–54	59	8
	55 years or older	35	4.7
Education	Bachelor’s Degree	460	62.1
	Completed high school (Tawjihi)	107	14.4
	Diploma	62	8.4
	Did not complete high school	58	7.8
	Postgraduate Degree (Master’s, PhD, etc.)	54	7.3
Occupation	Employed (Full or Part-time)	298	40.2
	Student	274	37
	Unemployed	148	20
	Retired	21	2.8
Residence	Urban (City/Major Town)	454	61.3
	Rural (Village/Camp)	287	38.7
Chronic disease	No	651	87.9
	Yes	90	12.1
Eye exam	Yes	481	64.9
	No	260	35.1
Heard glaucoma	Yes	400	54
	No	341	46
Screening importance	Yes, it is very important	569	76.8
	I am unsure	137	18.5
	No, I do not think it is necessary	35	4.7
Follow advice	I would definitely go	496	66.9
	I would probably go	198	26.7
	I would probably not go	26	3.5
	I would definitely not go	21	2.8
Last checkup	Within the last 1 year	199	26.9
	More than 3 years ago	189	25.5
	1 to 3 years ago	179	24.2
	Never	174	23.5

Item-level performance on the eight knowledge statements showed substantial variability, with frequent “Not sure” responses (**Table 2**). The highest correct response proportion was observed for the statement that early treatment can help prevent further vision loss (64.4% correct), followed by recognition that untreated glaucoma can cause irreversible blindness (57.0% correct) and that diabetes increases glaucoma risk (55.1% correct). Approximately half of respondents correctly identified that glaucoma affects the optic nerve (50.1%) and that adults aged ≥40 are at increased risk (49.0%), while fewer correctly identified family history as a risk factor (44.9%). The lowest correct response rates were observed for the two misconception statements requiring a “False” response: only 20.9% correctly rejected the statement that glaucoma usually has noticeable early symptoms such as pain/redness, and 20.9% correctly rejected the statement that glaucoma can be completely cured with full reversal of vision loss (**Table 2**). Item-level accuracy with 95% confidence intervals is illustrated in **Figure 1**.

**Table 2:** Distribution of responses to glaucoma knowledge items among participants (N = 741).

Item	Correct response	Correct (n)	Correct %	Incorrect (n)	Incorrect %	Not sure (n)	Not sure %
Glaucoma affects the nerve that connects the eye to the brain (the optic nerve)	True	371	50.1	38	5.1	332	44.8
Glaucoma usually has noticeable symptoms (like pain or redness) in its early stages	False	155	20.9	213	28.7	373	50.3
If left untreated, Glaucoma can cause permanent (irreversible) blindness	True	422	57	30	4	289	39
Glaucoma can be completely cured (vision loss can be fully reversed)	False	155	20.9	227	30.6	359	48.4
Receiving early treatment for Glaucoma can help prevent further vision loss	True	477	64.4	15	2	249	33.6
People aged 40 years or older are at a higher risk of developing glaucoma.	True	363	49	42	5.7	336	45.3
Having chronic diseases like diabetes increases a person’s risk of glaucoma.	True	408	55.1	26	3.5	307	41.4
Having a family member with glaucoma increases a person’s risk.	True	333	44.9	49	6.6	359	48.4

**Figure 1:**
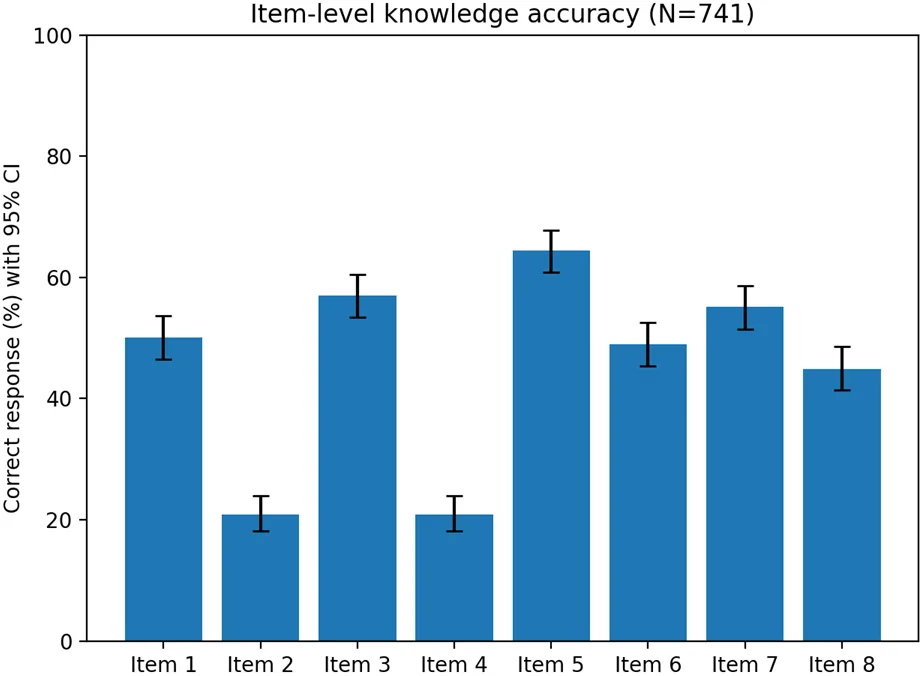
Proportion of correct responses for each glaucoma knowledge item with 95% confidence intervals (N = 741).

The total knowledge score ranged from 0 to 8, with a mean of 3.62 ± 2.74 and a median of 4 (IQR 0–6). A substantial proportion of respondents scored at the lower end of the scale, including 201 (27.1%) participants who scored 0, while 46 (6.2%) achieved the maximum score of 8. The score distribution is presented in **Figure 2**, and score patterns stratified by gender and residence are displayed in **Figure 3**. Internal consistency of the 8-item knowledge scale was high (Cronbach’s α = 0.87), supporting the reliability of the composite score.

**Figure 2:**
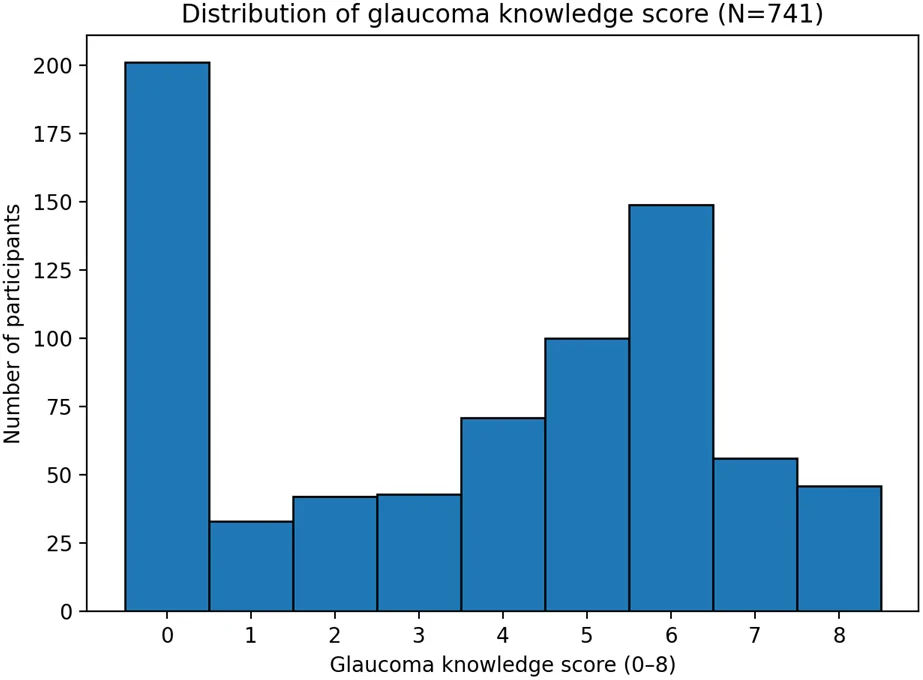
Distribution of total glaucoma knowledge scores among participants (N = 741).

**Figure 3:**
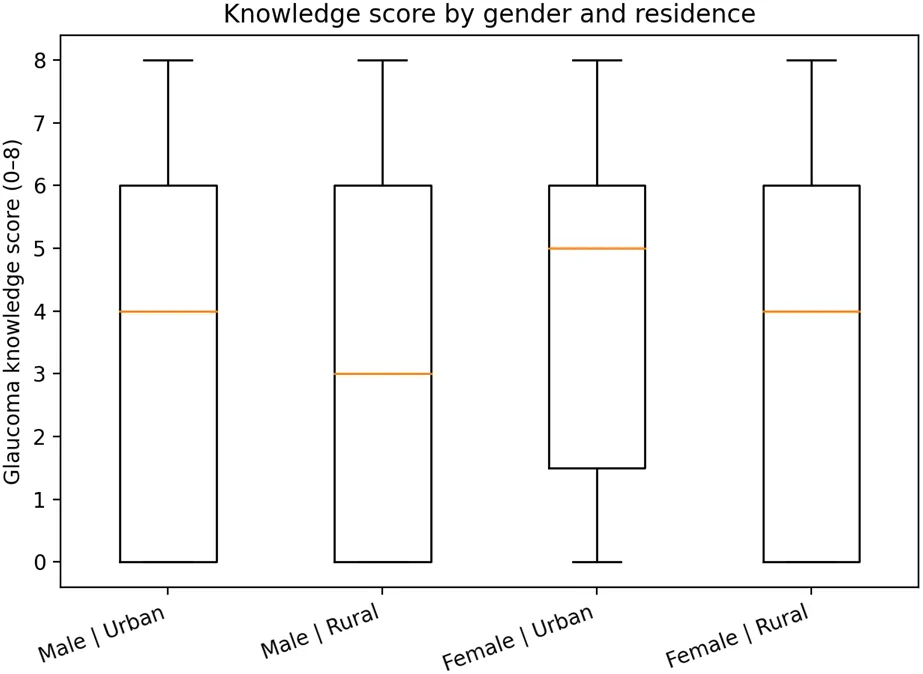
Glaucoma knowledge scores by gender and residence (N = 741).

To evaluate the robustness of associations to alternative “good knowledge” thresholds, two score-based sensitivity definitions were examined: Good75 (score ≥6/8, ≥75% correct) and strict good knowledge (Good80 (score ≥7/8, ≥87.5% correct)). Under these definitions, 33.9% of participants met the Good 75threshold, and 13.8% met the Strict good knowledge threshold. Bivariate comparisons using the Good 80.

Definitions are presented in **Table 3**, which includes multiple demographic, residence, clinical, and practice/attitude variables (including those not statistically significant, as specified). Using χ² testing, Good80 prevalence differed across age groups (overall p = 0.043) and education level (overall p = 0.011) and was higher among urban compared with rural residents (p = 0.012). Gender was not associated with strict good knowledge (p = 0.880), and chronic disease history was not associated with strict good knowledge in bivariate analysis (**Table 3**).

**Table 3:** Bivariate associations between participant characteristics and high glaucoma knowledge (Good80) (N = 741).

Predictor	Category	Good (n,%)	Poor (n,%)	χ² x2	p-value
Gender	Female	52 (14.1)	317 (85.9)	0.02	0.880
	Male	50 (13.4)	322 (86.6)	0.02	0.880
Age group	18-24	63 (17.0)	307 (83.0)	9.86	0.043
	25-34	24 (13.6)	152 (86.4)	9.86	0.043
	35–44	9 (8.9)	92 (91.1)	9.86	0.043
	45–54	3 (5.1)	56 (94.9)	9.86	0.043
	55 years or older	3 (8.6)	32 (91.4)	9.86	0.043
Education	Bachelor’s Degree	76 (16.5)	384 (83.5)	12.96	0.011
	Completed high school	6 (5.6)	101 (94.4)	12.96	0.011
	Did not complete high school	5 (8.6)	53 (91.4)	12.96	0.011
	Diploma	5 (8.1)	57 (91.9)	12.96	0.011
	Postgraduate Degree	10 (18.5)	44 (81.5)	12.96	0.011
Occupation	Employed (Full- or Part-time)	31 (10.4)	267 (89.6)	16.77	< 0.001
	Retired	5 (23.8)	16 (76.2)	16.77	<0.001
	Student	54 (19.7)	220 (80.3)	16.77	<0.001
	Unemployed	12 (8.1)	136 (91.9)	16.77	<0.001
Residence	Rural (Village/Camp)	25 (8.7)	262 (91.3)	9.4	0.002
	Urban (City/Major Town)	77 (17.0)	377 (83.0)	9.4	0.002
Chronic disease	No	93 (14.3)	558 (85.7)	0.89	0.346
	Yes	9 (10.0)	81 (90.0)	0.89	0.346
Eye exam	No	31 (11.9)	229 (88.1)	0.92	0.338
	Yes	71 (14.8)	410 (85.2)	0.92	0.338
Heard glaucoma	No	11 (3.2)	330 (96.8)	57.48	<0.001
	Y Yes	91 (22.8)	309 (77.2)	57.48	<0.001
Screening importance	I am unsure	1 (0.7)	136 (99.3)	27.85	<0.001
	No, I don’t think it is necessary	2 (5.7)	33 (94.3)	27.85	<0.001
	Yes, it is very important	99 (17.4)	470 (82.6)	27.85	<0.001
Follow advice	I would definitely go	81 (16.3)	415 (83.7)	10.5	0.015
	I would definitely not go	0 (0.0)	21 (100.0)	10.5	0.015
	I would probably go	20 (10.1)	178 (89.9)	10.5	0.015
	I would probably not go	1 (3.8)	25 (96.2)	10.5	0.015
Last checkup	1 to 3 years ago	31 (17.3)	148 (82.7)	7.64	0.054
	More than 3 years ago	25 (13.2)	164 (86.8)	7.64	0.054
	Never	14 (8.0)	160 (92.0)	7.64	0.054
	Within the last 1 year	32 (16.1)	167 (83.9)	7.64	0.054
Score cat	0–2	0 (0.0)	276 (100.0)	230.91	<0.001
	3–5	0 (0.0)	214 (100.0)	230.91	<0.001
	6–8	102 (40.6)	149 (59.4)	230.91	<0.001

Variables reflecting awareness and care-seeking behaviors demonstrated strong bivariate associations with strict good knowledge. Participants who had heard of glaucoma had markedly higher strict good knowledge prevalence than those who had not (p < 0.001). The perceived importance of screening for adults over 40 showed a particularly strong association with strict good knowledge (p < 0.001), and both the likelihood of following a provider’s referral advice and the timing of the last comprehensive eye check-up were associated with strict good knowledge (overall p < 0.001 for each; **Table 3**).

Multivariable logistic regression was performed to estimate adjusted associations with good knowledge using both sensitivity outcomes (Good75 and strict good knowledge). Adjusted effect estimates for both models are reported in **Table 4**. Prior awareness of glaucoma remained the strongest independent correlate of good knowledge in both models (Good75: aOR 7.89, 95% CI 5.08–12.25, p < 0.001; strict good knowledge: aOR 6.68, 95% CI 3.37–13.25, p < 0.001). In addition, uncertainty or non-endorsement regarding screening importance for adults over 40 was independently associated with substantially lower odds of good knowledge compared with endorsing screening as “very important” (Good75: aOR 0.08, 95% CI 0.03–0.22, p < 0.001; strict good knowledge: aOR 0.06, 95% CI 0.01–0.46, p = 0.006). Under the stricter strict good knowledge definition, urban residence was independently associated with higher odds of good knowledge (aOR 2.10, 95% CI1.24–3.58, p = 0.006). Education retained evidence of association in the strict good knowledge model, with completed high school (Tawjihi) showing lower adjusted odds of strict good knowledge compared with a bachelor’s degree (aOR 0.38, 95% CI 0.15–0.98, p = 0.045). Older age (≥55 years) was associated with lower odds of good knowledge compared with 18–24 years in both sensitivity models (Good75: aOR 0.24, 95% CI 0.06–0.94, p = 0.040; strict good knowledge: aOR 0.06, 95% CI0.01–0.66, p = 0.021).

**Table 4:** Multivariable logistic regression analysis of factors associated with glaucoma knowledge (Good75 and Good80) (N = 741).

Predictor	Category (vs reference)	aOR (≥6/8)	CI (≥6/8)	p (≥6/8)	aOR (≥7/8)	CI (≥7/8)	p (≥7/8)
Age group	25-34	1.26	0.71–2.22	0.433	1.05	0.53–2.09	0.891
	35–44	1.02	0.52–2.00	0.960	0.53	0.21–1.35	0.185
	45–54	1.12	0.50–2.50	0.784	0.14	0.03–0.72	0.019
	55 years or older	0.24	0.06–0.94	0.040	0.06	0.01–0.66	0.021
Chronic disease	Yes	1.5	0.82–2.75	0.189	0.85	0.36–2.05	0.722
Education	Completed high school (Tawjihi)	0.57	0.31–1.05	0.072	0.38	0.15–0.98	0.045
	Did not complete high school	1.15	0.53–2.51	0.727	1.12	0.38–3.29	0.832
	Diploma	1.02	0.51–2.07	0.947	0.93	0.32–2.69	0.893
	Postgraduate Degree (Master’s, PhD, etc.)	1.4	0.68–2.86	0.359	1.69	0.70–4.07	0.244
Eye exam	Yes	1	0.66–1.51	0.997	0.78	0.47–1.31	0.345
Gender	Female	1.51	1.02–2.23	0.039	1.27	0.78–2.06	0.343
Heard glaucoma	Yes	7.89	5.08–12.25	<0.001	6.68	3.37–13.25	<0.001
Occupation	Retired	2.24	0.57–8.88	0.251	15.14	1.99–115.01	0.009
	Student	2.11	1.21–3.69	0.009	1.65	0.83–3.29	0.153
	Unemployed	0.74	0.42–1.28	0.281	1.23	0.56–2.67	0.606
Residence	Urban (City/Major Town)	1.38	0.93–2.04	0.113	2.1	1.24–3.58	0.006
Screening importance	I am unsure	0.08	0.03–0.22	<0.001	0.06	0.01–0.46	0.006
	No, I do not think it is necessary	0.4	0.16–0.98	0.045	0.27	0.06–1.21	0.088

Finally, a strict sensitivity definition was evaluated to reduce misclassification due to misconception endorsement. Under this definition, participants were classified as “strict good knowledge” only if they scored ≥7/8 and answered both misconception items correctly (rejecting early pain/redness symptoms and rejecting “complete cure”). Using this criterion, 60/741 (8.1%) participants met the strict definition (reported in the strict sensitivity outputs). In the reduced multivariable model (constructed to mitigate sparse-data instability due to the low event rate), prior awareness of glaucoma remained independently associated with strict good knowledge (aOR 5.88, 95% CI 2.42–14.29, p < 0.001), and urban residence was also associated (aOR 2.13, 95% CI 1.11–4.11, p = 0.024). Lower odds of strict good knowledge were observed among participants who did not endorse screening importance (not sure/not necessary vs. very important: aOR 0.08, 95% CI 0.01–0.59, p = 0.014).

## Discussion

The aim of this population-based cross-sectional study is to determine the awareness and knowledge about glaucoma in the general population of Palestine. To our knowledge, this is the first study to investigate awareness and knowledge of glaucoma in the general population of Palestine.

In this study, 54% had ever heard of glaucoma. This level of awareness is higher than that reported in studies from neighboring countries. A study among the general population in Jordan reported an awareness level of 38% (12), while a cross-sectional study in Riyadh City, Saudi Arabia, found that 37.8% of participants had heard of glaucoma (13).

However, while more than half of respondents had heard the name of the disease, only 13.8% had strict good knowledge (scoring ≥7/8 on the knowledge scale). Studies from Jordan and Riyadh reported higher rates, with 47.6% of Jordanian participants having moderate to good awareness (2) and 28% of Riyadh participants having good awareness (with just 4.4% having excellent awareness) (13), both higher than our findings, though direct comparison is limited by different assessment criteria. This finding is comparable to a recent German population study by Terheyden et al., where only 9% of respondents correctly answered all glaucoma-related knowledge items (14).

The strongest predictor of good knowledge in our study was prior awareness of glaucoma, an expected association based on previous scoping review in the literature (15). Participants who had previously heard of glaucoma were approximately eight times more likely to have good knowledge compared to those who had not heard of the disease (aOR=7.89, P < 0.001). However, even among those who had heard of glaucoma, only 22.8% had good knowledge scores. This finding is consistent with studies from Saudi Arabia (13) and Ethiopia (16), where prior awareness of glaucoma was significantly associated with better knowledge levels.

Our study revealed significant gaps in specific knowledge aspects that are critical for early detection, particularly regarding the asymptomatic nature of the disease, irreversibility of blindness, and knowledge of risk factors.

Glaucoma is often called the silent thief of sight because major optic nerve damage can occur before it causes any symptoms (17). In our study, only 20.9% of participants correctly recognized that glaucoma is asymptomatic in its early stages. Nearly one-third (28.7%) of participants believed that glaucoma presents with noticeable symptoms such as pain or redness early in the disease course.

The difficulty in recognizing the asymptomatic nature of early glaucoma has also been documented across diverse populations, suggesting that this is a persistent and internationally relevant knowledge gap rather than a finding unique to Palestine. In a large survey of rural residents in North India, Rewri and Kakkar identified substantial deficiencies in public knowledge regarding glaucoma and its early clinical course (18). Similarly, a representative German population survey found that respondents frequently attributed glaucoma to symptoms such as blurred vision and pain, while overall knowledge of its signs, symptoms, and course was poor (19). More recently, the Italian KED-IT questionnaire study identified knowledge of major eye diseases, including glaucoma, as an important component of public eye-health literacy and emphasized the need for reliable assessment of disease-specific knowledge (20). In the Maldives, only 13.1% of participants correctly recognized that glaucoma may have an asymptomatic course, while 68.5% were unaware of this characteristic (21). Although differences in study populations and questionnaires limit direct quantitative comparisons, these findings are broadly consistent with our result that only 20.9% of Palestinian participants correctly recognized the absence of noticeable symptoms in early glaucoma. This recurring misconception is clinically important because reliance on pain, redness, or other noticeable symptoms may create a false sense of reassurance and delay appropriate eye examinations, particularly among individuals at increased risk. Public education should therefore explicitly communicate that glaucoma may progress without noticeable symptoms and that detection cannot depend on symptom recognition alone.

In the present study, 57% correctly recognized that untreated glaucoma causes permanent blindness, comparable to findings from Jordan, where 40.5% identified glaucoma as a blinding condition (10). However, only 20.9% in our sample identified that glaucoma cannot be completely cured, much lower than the 52.2% of Jordanian participants who correctly identified that glaucoma is treatable but not curable (10).

Recognition of glaucoma risk factors was limited in the present study. Age ≥40 years was correctly identified as a risk factor by 49.0% of participants, family history by 44.9%, and diabetes by 55.1%. Diabetes was the better-recognized risk factor among the three, likely reflecting broader public health awareness about diabetic complications.

The presence of chronic disease, including diabetes, was not significantly associated with better glaucoma knowledge (p=0.346), despite 12.1% of participants having chronic conditions. This finding contrasts with a study from the western region of Saudi Arabia, where participants with diabetes had significantly higher awareness rates (70.2% compared with 64.4%) (21). However, when examining detailed knowledge scores rather than basic awareness, the same study found no significant association between diabetes and knowledge, similar to our findings.

Participants aged 55 years and older had the poorest knowledge levels, with significantly lower odds of having good knowledge compared to younger age groups (aOR=0.24, p=0.040). This pattern was also seen in studies from Saudi Arabia (13) and Ethiopia (16), where younger participants had better glaucoma knowledge than older age groups.

Consistent with previous findings from studies in Saudi Arabia (13) and Ethiopia (16), the present study identified a statistically significant association between educational level and glaucoma knowledge. Participants with postgraduate and bachelor’s degrees had the highest rates of good knowledge (18.5% and 16.5%, respectively), whereas those with a high school education or less showed lower understanding of the disease (5.6%). The lower level of knowledge observed among elderly participants in our study may partly reflect the lower educational attainment in this age group compared to younger populations.

In the present study, retired participants and students had the highest rates of good knowledge (23.8% and 19.7%, respectively), followed by employed individuals (10.4%), while unemployed participants had the poorest understanding (8.1%). In the multivariate analysis of the study results, students were more likely to have good knowledge compared to employed individuals. This association may reflect greater engagement with educational environments, increased use of digital information sources, and higher health literacy among students, even after adjusting for other sociodemographic factors.

In contrast to education, occupation, and age, gender showed minimal association with glaucoma knowledge in bivariate analysis (p=0.880), with 14.1% of females and 13.4% of males having good knowledge. After adjusting for confounders, female gender showed a modest positive association (aOR=1.51, p=0.039), though this effect was weaker than other predictors and disappeared at strict knowledge cutoffs. This finding contrasts with studies from neighboring countries. In Saudi Arabia’s Riyadh region, gender was significantly associated with glaucoma awareness, with women having better knowledge levels (13). The small gender difference in our study may reflect the relatively high proportion of educated participants (62.1% with bachelor’s degrees) and urban residents (61.3%), populations in which access to education and health-related information is likely more equitable between genders.

Residents of urban centers had almost twice the rate of good knowledge (17%) compared to those in rural villages and camps (8.7%). This gap may reflect the concentration of healthcare services and public health campaigns in cities, with rural populations having less access to health information. This finding is consistent with results found in studies from northwest Ethiopia (16) and southern India (22).

The inadequate level of glaucoma knowledge represents a major barrier to effective screening behavior in Palestine. Early detection is fundamental in glaucoma management, as the disease is asymptomatic in early stages when intervention is most effective (17). This knowledge gap is reflected in our findings regarding eye examination behavior. Nearly one-quarter of participants (23.5%) reported never having undergone any eye examination, and only 26.9% had been examined within the preceding year.

Having previously undergone an eye examination was not significantly associated with better glaucoma knowledge (p=0.338), suggesting that routine clinical visits do not include adequate glaucoma education. Participants who would definitely go for screening if advised had better knowledge (16.3%) compared with those who would “probably not go” (3.8%) or “definitely not go” (0%), indicating a significant association between screening intention and knowledge (p=0.015).

Our findings have several implications for glaucoma prevention in Palestine. Although over half of the participants recognized the term “glaucoma,” fewer than one in seven understood key aspects of the disease. This suggests that awareness campaigns alone are not enough. Education must move beyond name recognition to address the asymptomatic nature of early disease, the irreversibility of vision loss, and established risk factors. The lack of association between previous eye examinations and knowledge levels suggests that clinical visits are not being used adequately to educate patients. Training ophthalmologists and primary care physicians to routinely discuss glaucoma during consultations may improve population knowledge. Knowledge gaps were largest among older adults, rural residents, and unemployed individuals. These groups may be at higher risk of visual impairment or blindness because of their limited awareness/knowledge about the disease. Prevention strategies should focus on reaching these populations through community health workers, integration into primary care services, and mobile screening programs.

The study had several strengths. The large sample size (n=741) gave adequate statistical power to find associations between demographic factors and knowledge. The eight-item knowledge assessment covered multiple aspects of glaucoma (pathophysiology, symptoms, outcomes, and risk factors), allowing us to identify specific gaps. Including both urban and rural participants across different age groups and educational levels makes the findings more generalizable to the Palestinian population.

However, several limitations should be noted. Self-reported data on chronic disease and eye examination history may be inaccurate. The use of non-probability voluntary response sampling through a predominantly online questionnaire introduces an important risk of selection bias and limits the representativeness of the sample. The study population was disproportionately composed of younger adults (49.9% aged 18–24 years), participants with a bachelor’s degree (62.1%), and urban residents (61.3%), who may have greater access to digital information, higher health literacy, and greater engagement with health-related content than the broader Palestinian adult population. Because these characteristics were also associated with glaucoma knowledge in our analysis, the sampling structure likely biased the observed knowledge levels upward and may have underestimated the magnitude of knowledge gaps among older adults, rural residents, and individuals with lower educational attainment. However, the exact magnitude of this bias cannot be quantified because probability-based population sampling and population-representative weighting were not used. Accordingly, the observed prevalence of adequate glaucoma knowledge should not be interpreted as a population-representative estimate for Palestinian adults. The age-, education-, and residence-related differences observed within the sample should instead be interpreted as internal associations that warrant confirmation in future population-based studies using probability sampling and appropriate population weighting.

## Conclusion

This study highlights substantial gaps in public knowledge and awareness of glaucoma in Palestine. While over half of participants had heard of glaucoma, only a small fraction possessed adequate knowledge regarding its asymptomatic progression, irreversibility, and risk factors. Prior awareness, younger age, higher education, urban residency, and student status were significantly associated with better knowledge. The findings indicate that awareness campaigns alone are insufficient; targeted educational interventions integrated into primary care and community programs are essential to improve early detection and screening uptake and ultimately reduce glaucoma-related blindness. According to our results, to address health disparities in glaucoma awareness, we should give special attention to older adults, rural residents, and those with lower educational levels.

## Data Availability

The raw data supporting the conclusions of this article will be made available by the authors, without undue reservation.
